# Exploring the transcriptome of immature stages of *Ornithodoros hermsi*, the soft-tick vector of tick-borne relapsing fever

**DOI:** 10.1038/s41598-024-62732-6

**Published:** 2024-05-30

**Authors:** Lucas C. de Sousa-Paula, Markus Berger, Octavio A. C. Talyuli, Cindi L. Schwartz, Greg A. Saturday, José M. C. Ribeiro, Lucas Tirloni

**Affiliations:** 1 Tick-Pathogen Transmission Unit, Laboratory of Bacteriology, Rocky Mountain Laboratories, Division of Intramural Research, National Institute of Allergy and Infectious Diseases, Hamilton, MT USA; 2 Grupo de Reprodução e Farmacologia Celular, Laboratório de Bioquímica Farmacológica, Centro de Pesquisa Experimental (CPE), Hospital de Clínicas de Porto Alegre (HCPA-UFRGS), Porto Alegre, RS Brazil; 3 Mosquito Immunity and Vector Competence Section, Laboratory of Malaria and Vector Research, Division of Intramural Research, National Institute of Allergy and Infectious Diseases, Rockville, MD USA; 4 Electron Microscopy Unit, Research Technologies Branch, Rocky Mountain Laboratories, Division of Intramural Research, National Institute of Allergy and Infectious Diseases, Hamilton, MT USA; 5 Rocky Mountain Veterinary Branch, Rocky Mountain Laboratories, Division of Intramural Research, National Institute of Allergy and Infectious Diseases, Hamilton, MT USA; 6 Vector Biology Section, Laboratory of Malaria and Vector Research, Division of Intramural Research, National Institute of Allergy and Infectious Diseases, Rockville, MD USA

**Keywords:** Transcriptomics, Entomology

## Abstract

Blood-feeding behavior has independently evolved in arthropods multiple times. Unlike hard ticks, soft ticks employ a rapid-feeding strategy for hematophagy, and there are comparatively limited studies on the transcriptomes of these organisms. This study investigates the soft tick *Ornithodoros hermsi*, conducting histopathological examinations at bitten skin sites and tick whole-body transcriptomic analyses across various developmental and feeding stages, including larvae, 1st-nymphal, and 2nd-nymphal stages. The results revealed the ability of *O. hermsi* to induce skin hemorrhage at the bite sites. Transcriptomic analyses identified three consistent transcriptional profiles: unfed, early-fed (6 h, 12 h, 24 h), and late-fed (5 days). The unfed profile exhibited high transcriptional activity across most of the functional classes annotated. In contrast, early-fed stages exhibited decreased expression of most functional classes, except for the unknown, which is highly expressed. Finally, transcriptional expression of most functional classes increased in the late-fed groups, resembling the baseline expression observed in the unfed groups. These findings highlight intense pre-feeding transcriptional activity in *O*. *hermsi* ticks, aligning with their rapid-feeding strategy. Moreover, besides shedding light on the temporal dynamics of key pathways during blood meal processing and tick development, this study contributes significantly to the transcriptome repertoire of a medically relevant soft tick species with relatively limited prior knowledge.

## Introduction

Hematophagous behavior has independently evolved more than 20 times in the phylum Arthropoda^1^. Evolutionary processes have driven ticks to become obligate blood feeders^2^. Remarkably, blood-feeding strategies evolved differently between the two main families of ticks. Hard ticks (Ixodidae) are slow feeders, remaining attached to hosts for days or weeks until they complete a blood meal. In contrast, soft ticks (Argasidae) exhibit a generally fast-feeding strategy by making brief and intermittent visits to their hosts to obtain a blood meal. These distinctions, alongside variations in the number of hosts, host preferences, mating behaviors, and more, underscore the remarkable diversity within the tick group^3^.

*Ornithodoros hermsi* Wheeler is an important soft-tick species because of its role as a vector of *Borrelia hermsii* and *Candidatus* Borrelia nietonii, the bacteria causing tick-borne relapsing fever (TBRF) in the western United States^4,5^. This tick is nidicolous, with a wrinkled body, lacks a scutum, and possesses relatively short mouthparts, which are located on the underside of the body. Each developmental stage exhibits unique physiological characteristics. Its lifecycle includes eggs, larval stage, 2 to 5 nymphal instars (primarily 3), and adults, with females being slightly larger than males^6,7^. *Ornithodoros hermsi* exhibits a rapid feeding pattern in all life stages, lasting from 15 to 90 min. Females lay small batches of eggs after each blood meal and can have several gonotrophic cycles during their lifetime^8^. Despite being a tick of medical importance in the United States, little is known about the molecular mechanisms employed by *O. hermsi* during blood meal and blood digestion.

Applications of “omic” technologies have facilitated the identification of a large set of tick molecules that impact tick feeding and blood digestion, which play a role in the transmission of tick-borne pathogens^9,10^. Assembling and annotating the identified transcripts and describing the transcriptomes and putative proteomes of various tick species contribute to our understanding of tick physiology, feeding biology, and evolution^11^. Several previous studies have characterized transcriptomes of several hard tick species, reviewed in ^9–11^. Comparing to hard ticks, only a few studies have focused on understanding mechanisms underlying the hematophagy strategy of soft ticks^12–18^.

To gain insights into the blood feeding strategies employed by *O*. *hermsi* ticks, the effect of *O*. *hermsi* tick bites on the skin of neonate mice was analyzed. Additionally, a comprehensive analysis of the whole-body transcriptome composition in immature stages (larval, 1st-nymphal, and 2nd-nymphal stages) was performed. By analyzing unfed and fed groups at different timepoints, we explored the molecular mechanisms behind the fast-feeding strategy used by this tick species during blood meal and blood digestion.

## Methods

### The Ethics statement

Animal experiments were conducted in accordance with the guidelines of the National Institutes of Health on protocols approved by the Rocky Mountain Laboratories Animal Care and Use Committee (2021-060) and in compliance with the recommendations of the ARRIVE guidelines. The Rocky Mountain Veterinary Branch is accredited by the International Association for Assessment and Accreditation of Laboratory Animal Care (AAALAC).

### Ticks and tick feeding

The *O. hermsi* ticks used in this study were obtained from a colony maintained at Rocky Mountain Laboratories in Hamilton, MT. This colony originated from a single, uninfected, fertilized female collected in Siskiyou County, California, in 1997^19^. The ticks were kept in environmental incubators at 21 °C and approximately 85% relative humidity before being fed on neonate mice (RML strain). For feeding, neonate mice were placed into plastic, round, clear jar with screw-capped lids. These lids were perforated in the middle with an opening covered by nylon mesh screens, and they had a thick plaster-of-Paris lining on the bottom^20^. We choose to utilize larvae, 1st-nymphal, or 2nd-nymphal stages due to specimen availability in our colony. Additionally, as *O*. *hermsi* 2nd-nymphs might molt into adults after a blood meal, we considered these three stages representative of the immature phase. Unfed larvae, 1st-nymphal, or 2nd-nymphal stages ticks were placed in these chambers which rested in the dark at room temperature. Mice were checked periodically, and fully engorged ticks were collected and stored in 15 mL plastic tubes containing strips of filter paper to absorb coxal fluid. These tubes also had perforated caps for ventilation. Finally, the tubes were kept in environmental incubators as described above.

### Histopathology of bitten mouse skin

After completion of tick feeding (up to 2 h after tick placement), neonate mice were euthanized by isoflurane overdose followed by decapitation. Skin bitten sites were collected using a 2 mm biopsy punch and immediately placed in 10% neutral buffered formalin solution. Histopathological investigation consisted of macroscopic and microscopic examination of skin bitten sites and standard hematoxylin–eosin staining in thin sections embedded in paraffin blocks, followed by a histopathological scoring performed by a pathologist.

### Library preparation, sequencing, and data analysis

Total RNA was extracted from whole-body *Ornithodoros hermsi* larvae, 1st-nymphal, and 2nd-nymphal stages. The ticks were grouped into three biological replicates, each consisting of approximately fifty (50) larvae, ten (10) nymphs at the 1st-nymphal stage, and five (5) nymphs at the 2nd-nymphal stage per replicate. These groups included unfed ticks (UF) and ticks fed on neonate mice at different time points: 6 h post-detachment (FED6h), 12 h post-detachment (FED12h), 24 h post-detachment (FED24h), and 5 days post-detachment (FED5d). RNA extraction was carried out using the PureLink™ RNA Mini Kit (Invitrogen, Waltham, USA) following the manufacturer's specifications. Subsequently, RNA quality was assessed using an Agilent High Sensitivity RNA ScreenTape System on a TapeStation 4150 (Agilent, Santa Clara, USA). Library construction was performed using the NEBNextUltraTM II (Directional) RNA with polyA selection library prep kit. The sequencing was performed using an Illumina NovaSeq 6000 (PE150), with an estimated 40 million paired-end reads per sample.

Low-quality sequences with a Phred quality score (Q) below 20 were removed and the Illumina adaptors were trimmed using TrimGalore (https://github.com/FelixKrueger/TrimGalore). Subsequently, reads were merged and de novo assembled using Trinity (2.9.0)^21^, in single-stranded F mode, and ABySS (2.3.1)^22^ with k values ranging from 25 to 95, with increments of 10. The final assemblies were merged, and sequences sharing at least 95% identity were consolidated using the CD-HIT tool^23^.

The DNA coding sequences (CDS) with an open reading frame (ORF) of at least 100 nucleotides were extracted based on BLASTp results from several databases, including subsets of the non-redundant and transcriptome shotgun assembly from National Center for Biotechnology Information (NCBI) databases (chelicerata, Rickettsiales, and Borreliaceae), RefSeq invertebrate (https://ftp.ncbi.nlm.nih.gov/refseq/release/invertebrate/), RefSeq protozoa (https://ftp.ncbi.nlm.nih.gov/refseq/release/protozoa/), RefSeq mitochondrion (https://ftp.ncbi.nlm.nih.gov/refseq/release/mitochondrion/), tick genomes^24^, mouse genome, and virus-phage. The CDS were extracted if they covered at least 70% of a matching protein. Additionally, all ORFs starting with a methionine and with a length of at least 40 amino acids were subjected to the SignalP tool (V3.0)^25^. Sequences with a putative signal peptide were mapped to the ORFs, and the most 5’ methionine was selected as the starting point of the transcript. Relative quantification of CDS was performed by mapping the library of adapter-trimmed reads to the extracted CDS using the RSEM tool^26^. Functional annotation of the selected CDS was carried out using an *in-house* program that scanned a vocabulary of approximately 400 words and their order of appearance in the protein matches obtained from BLASTp/RPS-BLAST against various databases, including chelicerata from NCBI (https://www.ncbi.nlm.nih.gov/genbank/) and Uniprot (https://www.ebi.ac.uk/uniprot/), RefSeq invertebrate (https://ftp.ncbi.nlm.nih.gov/refseq/release/invertebrate/), RefSeq vertebrate (https://ftp.ncbi.nlm.nih.gov/refseq/release/vertebrate/), RefSeq protozoa (https://ftp.ncbi.nlm.nih.gov/refseq/release/protozoa/), RefSeq mitochondrion (https://ftp.ncbi.nlm.nih.gov/refseq/release/mitochondrion/), tick genomes^24^, Rickettsiales and Borreliaceae from NCBI, uniprotkb, SMART, CDD, PFAM, KOG, EC, and COG (ftp://ftp.ncbi.nih.gov/pub/mmdb/cdd/little_endian/), MEROPS (https://ftp.ebi.ac.uk/pub/databases/merops/current_release/seqlib/), and TickSialoFam^9^. Predictions of signal peptide, transmembrane domains, furin cleavage, GPI anchor, and glycosylation sites were determined with software from the Center for Biological Sequence Analysis (https://www.cbs.dtu.dk/services/). The final annotated CDS are available for download as a hyperlinked spreadsheet file. To assess assembly quality, the benchmark for universal single-copy orthologs (BUSCO; Arthropoda database) was used^27^.

### Statistical analysis

Differential expression analysis was conducted using the *edgeR* package^28^ in R. Statistical significance was considered when the log_2_ (fold change) was greater than 2 or lesser than –2, and the false discovery rate (FDR) was less than 0.05. The heatmap plot was generated using *gplots* package^29^, using the TPM values represented as Z-score, while the volcano plots were generated using the *ggplot2* package^30^.

### Electron microscopy of *O. hermsi* midguts

We used electron microscopy to prospect the presence of whorl-like rough endoplasmic reticulum (RER) in *O*. *hermsi* midgut epithelial cells. Excised tick midguts of 2nd-nymphal stage and adult females were carefully dissected in phosphate-buffered saline, pH 7.4 (PBS) and fixed with 2% paraformaldehyde + 2.5% glutaraldehyde in 0.1 M Sorenson’s phosphate buffer (PB). Samples were processed with a Biowave microwave oven (Ted Pella, Inc.). Briefly, samples were rinsed with 0. 1 M PB, fixed with 0.5% OsO_4_ + 0.8% K_4_Fe(CN)_6_ in a cycle of 2 min on, 2 min off run 5 times. Samples were rinsed with distilled water (dH_2_O) and the same cycle [5× (2 min on, 2 min off)] was performed with 1% aqueous tannic acid, rinsed with dH_2_O, and the same cycle [5× (2 min on, 2 min off)] with aqueous 1% uranyl acetate. Samples were rinsed with dH2O followed by dehydration in a graduated ethanol series and microwave assisted embedding into eponate with polymerization at 60 °C overnight. 70 nm sections were examined at 80 keV using an HT7800 transmission electron microscope (Hitachi High Technologies Inc.) and digital images were captured using XR81-B camera (AMT).

To ensure the procedures to visualize whorl-like RER in epithelial cells, we used mosquito midguts as a control. *Aedes aegypti* (Liverpool strain) were reared at the Laboratory of Malaria and Vector Research (LMVR/NIAID/NIH), with insectary facility at 28 °C, 80% humidity under a 12 h light/dark cycle. The larvae stages were fed with fish food (TetraMin Tropical tablets) and adult mosquitoes were kept with cotton balls soaked in 10% sucrose solution. Midguts of 30 sugar-fed mosquito females (5 days old) were dissected in PBS, pH 7.4 and fixed with 4% paraformaldehyde + 2.5% glutaraldehyde in 0.1 M cacodylate buffer pH 7.4. Samples were processed for electron microscopy as described above.

## Results and discussion

### The transcriptome of *O*. *hermsi* immature stages

Despite being a tick of medical importance in the United States, little is known about the molecular mechanisms employed by *O. hermsi* ticks during blood meal and blood digestion. As of February 13, 2024, a search on NCBI revealed only 39 coding sequences for this species, all of which are of mitochondrial origin. While previous studies on soft-tick species have analyzed tissue-specific transcriptomes, such as those of salivary gland and midgut tissues^12–18^, our research stands out by examining the transcriptional activity of the entire tick body, including comparative analyses of transcriptomes across different stages.

**Figure 1:**
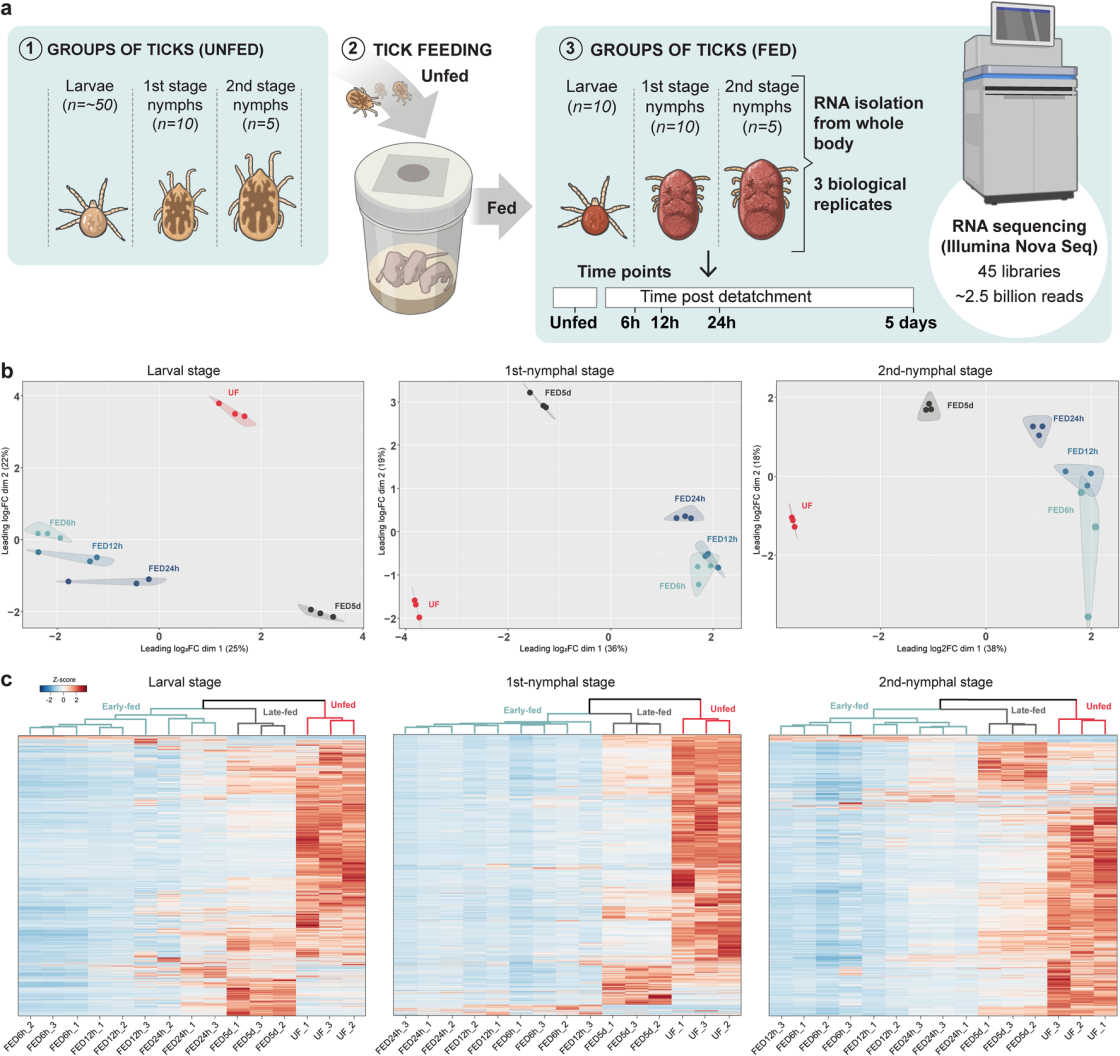
Overview of the transcriptional profile of *Ornithodoros hermsi* immature stages across different feeding timepoints. (**a**) Ticks were fed on neonate mice until blood repletion. Total RNA was isolated from the whole body of unfed (UF) and fed ticks at 6 h (FED6h), 12 h (FED12h), 24 h (FED24h), and 5 days (FED5d) post-blood feeding. (**b**) Multidimensional scaling (MDS) plots depict transcripts with transcripts per million (TPM) values ≥ 5 across all replicates within each triplicate. Groups are sorted based on transcriptional profiles, revealing three clusters: UF (red), early-fed (FED6h, FED12h, FED24h, blueish), and late-fed (FED5d, dark grey). (**c**) Heatmap plots comparing the normalized abundance of transcripts between feeding groups. Groups were clustered based on relative abundance (TPM ≥ 5) and transformed into Z-score. Each column represents the Z-score values for each replicate. Lines represent transcripts expressed by each replicate. Clades are color-coded to correspond to the MDS clusters.

A comprehensive set of 45 libraries was generated from both unfed (UF) and fed *O*. *hermsi* larval, 1st-nymphal, and 2nd-nymphal stages at four distinct timepoints: FED6h, FED12h, FED24h, and FED5d following blood feeding (Fig. 1a). This approach yielded a substantial dataset of over 2.4 billion paired-end reads. After excluding Illumina adapters, low-quality sequences, and utilizing a de novo assembly approach that combined results from Trinity and ABySS, followed by a CDS extraction pipeline, 103,646 potential CDS were extracted (Supplementary File 1). Subsequently, by removing potential contaminants from vertebrate or bacterial origins, 96,404 potential CDS were obtained. Employing the Benchmark Universal Single Copy-Orthologue (BUSCO) analysis, using the Arthropoda database as a reference, revealed a completeness assessment of 74.5% (58.1% single and 16.4% duplicate), 10.5% fragmented, and 15.0% missing.

Relative quantification of each CDS extracted was calculated by mapping the trimmed reads back from each library to the extracted CDSs, with an average read mapping rate ranging from 19 to 47%. This rate of unmapped reads was as expected for a de novo assembled transcriptome, given the absence of 5' and 3' untranslated regions (UTRs) and non-coding RNAs from the extracted CDS. For further functional annotation and differential expression analysis, we considered exclusively CDS displaying a TPM ≥ 5 in all three replicates of at least one of the biological groups. This selection process yielded a total of 18,465 CDS, which were then utilized for downstream analyses and discussion. This hyperlinked spreadsheet containing all annotated sequences is available in Supplementary File 2.

### *Ornithodoros hermsi* ticks display an intense transcriptional activity before blood feeding

Upon comparing the transcriptomes, three distinct clusters of transcriptional profiles were observed: unfed, early-fed (comprising FED6h, FED12h, and FED24h groups), and late-fed (FED5d). Notably, these same profiles were consistent across all developmental stages, including larval, 1st-nymphal, and 2nd-nymphal stages (Fig. 1b,c). To gain insights into the modulation of gene expression, we performed pairwise comparisons of transcripts differently expressed between feeding groups for all three developmental stages.

**Figure 2:**
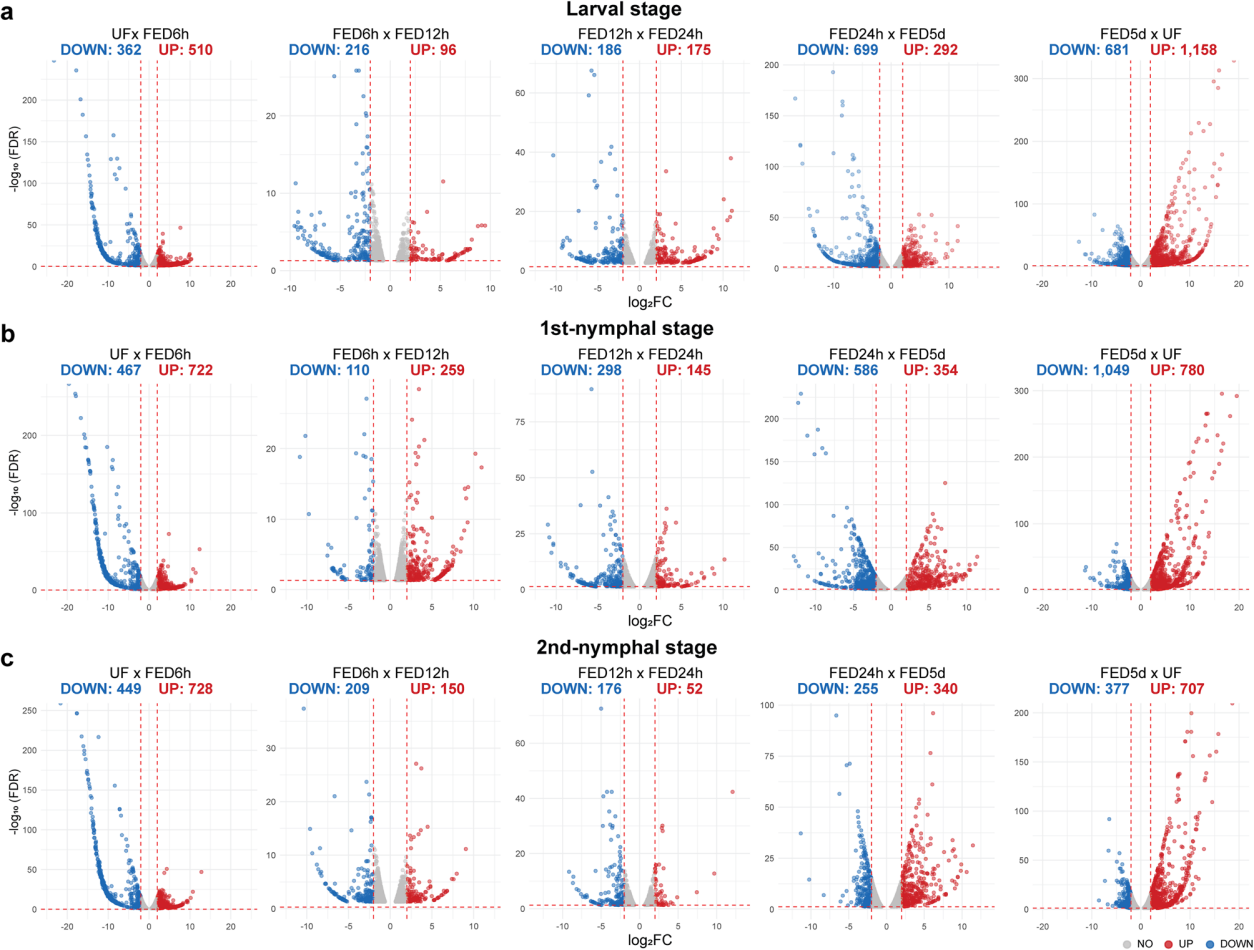
Comparison of transcript expression of *Ornithodoros hermsi* immature stages across different feeding timepoints. Volcano plots displaying differentially expressed transcripts between pairwise comparisons of different blood feeding groups for (**a**) larval, (**b**) 1st-nymphal, and (**c**) 2nd-nymphal stages. Statistical difference was considered when a transcript presented log_2_ fold change (log_2_FC) ≥ ± 2 (vertical dotted lines) and false discovery rate (FDR) ≤ 0.05 (horizontal dotted lines). Numbers above the plots indicate the number of transcripts upregulated (red) or down-regulated (blue). Transcripts that were not considered differentially expressed are shown as gray dots.

The first comparisons between UF and FED6h showed 872, 1,189, and 1,177 transcripts significantly modulated for larvae, 1st-nymphal, and 2nd-nymphal stages, respectively (Fig. 2). It was evident that blood-meal acquisition was followed by a high gene expression modulation in all three developmental stages, indicating a gene expression shift likely for blood digestion. Interestingly, comparisons between the timepoints within early-fed group showed considerable modulation of profile expression, varying from 228 to 443 transcripts differentially expressed. Even though these three timepoints have a similar gene expression profile (as depicted by MDS plots; Fig. 1b), this modulation reflects different events, as blood meal accommodation and digestion progresses, occurring during the first 24 h after feeding. Thereafter, the modulation of expression increased between FED24h and FED5d (ranging from 595 to 991 transcripts). Finally, the highest modulation was observed when comparing FED5d and UF, with 1839, 1829, and 1084 transcripts differently expressed for larvae, 1st-nymphal, and 2nd-nymphal stages, respectively. These differences underscore an intense transcriptional activity of a distinct set of genes, likely those important for final blood meal digestion and preparation for molting.

To gain further insights into how this translates to the *O. hermsi* physiology, we classified the final 18,465 CDS into 25 functional classes. Twenty-two out of twenty-five classes were highly expressed by unfed ticks. These functional classes comprise most of the essential components for accommodating the blood meal, preparing for its digestion, and consequent molting. Interestingly, all these classes exhibit almost the same pattern: they are highly expressed in unfed individuals, their expression drops drastically at 6 h after feeding, and then begins to increase as digestion progresses, apparently resembling the baseline expression in FED5d as observed in the unfed groups. These findings highlight a substantial shift in transcriptional activity among *O. hermsi* ticks throughout feeding. One plausible interpretation of these results is the rapid feeding strategy used by *O. hermsi* ticks. To successfully acquire and initiate the digestion of the blood meal, they need to transcribe essential genes related to these processes upfront, which will be ready for protein translation when feeding starts. The expression of these functional groups appears to return to the levels of unfed ticks on day 5, suggesting the resumption of gene expression important for the next feeding. This strategy ensures that the core genes required for successful blood feeding and digestion are already transcribed and ready to be translated (or already translated) as soon as they attach to a host. The fact that this pattern was observed in all stages analyzed here reinforces this hypothesis. It is important to note that although these functional groups appear to return to the levels of unfed ticks, by pairwise comparison, we have observed the highest modulation when comparing FED5d and UF (Fig. 2).

This observation suggests that different transcripts from the same functional classes are being expressed, which might represent a strategy to switch the molecular repertoire needed for the next developmental stage and a subsequent blood meal. It is important to note that further evidence at the protein level is necessary to validate this hypothesis.

**Figure 3:**
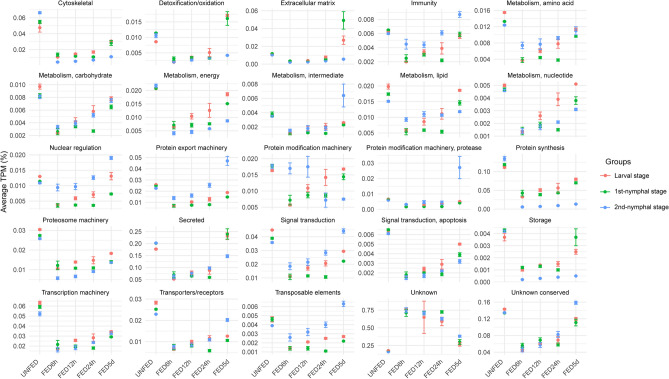
Relative quantification of functional classes of *Ornithodoros hermsi* immature stages across different feeding timepoints. The average TPM (%) of each class was plotted against each biological group. The error bars represent the standard deviation of the mean. Plots were generated using the *ggplot2* (3.4.2) package for R.

Our functional classification also includes the “unknown” and “unknown conserved” classes. The CDS within these functional groups are those exhibiting a high degree of similarity to previously deposited sequences of currently unknown function (“unknown conserved”) or displaying no or low similarities with previously deposited sequences (“unknown”), thereby representing potential novel sequences from ticks or specific to this tick species. Remarkably, these functional classes consistently emerged as the most abundant across all biological conditions, as shown in Supplementary File 3. The “unknown conserved” class follows the same pattern as observed for most classes, being highly expressed in unfed ticks, dropping after 6 h, and resembling the same level as unfed ticks after 5 days (Fig. 3). Notably, the transcripts from the “unknown” class have their expression induced by the blood meal. These transcripts have basal expression in unfed ticks, are upregulated at FED6h, maintain almost steady expression throughout FED24h, and then decline again at FED5D (Fig. 3). This observation suggests a critical role for these transcripts during blood feeding and underscores the overall knowledge gap in our understanding of tick physiology.

Based on above mentioned findings, we wondered which mechanism would be involved in this pattern of gene expression. In hematophagous dipterans (e.g., mosquitoes and sand flies), the rapid response to blood ingestion has been associated with the presence of large whorl-like RER in midgut epithelial cells^31,32^. It has been well-demonstrated in mosquitoes that whorls are remarkably present in unfed females, unfolding and expanding after the ingestion and during the digestion of blood, and then folding back^31,33–35^; this is absent in males (non-hematophagous)^36^. Presumably, this facilitates the synthesis and secretion of proteases into the mosquito digestive lumen after blood feeding. In fact, there has been demonstrated an intrinsic association between RNA molecules and the whorls^31^ and its formation has been shown to be dependent on alpha-COPI coatomer protein expression^37^. Interestingly, we observed homologous genes of the COPI coatomer complex (Supplementary File 2) with modulated expression over the time points analyzed. Most of them were highly expressed in the unfed groups, experienced drastic decreases during the FED6h and FED12h groups, and then showed increased expression at FED24h, persisting at FED5d. Although the presence of these genes initially hinted at the possibility of whorl-like RER, we conducted transmission electron microscopy (TEM) to verify the eventual presence of such ultrastructure. First, we used unfed mosquito midguts as positive controls to visualize the whorl-like RER. The presence of whorls was blindingly obvious (Supplementary Fig. 1). Then we repeated the same approach for unfed *O*. *hermsi* midguts. However, we did not observe whorls despite thorough screening (Supplementary Fig. 1). Similarly, exploring some images from another study focusing on *Ornithodoros moubata* midgut ultrastructure, we did not observe the presence of whorl-like RER^38^, suggesting it might be absent in soft ticks. The mechanisms contributing to the control of gene expression and protein translation in *Ornithodoros* ticks remains poorly understood and deserves further investigation.

### Genes highly transcribed in late fed ticks: A core of salivary genes?

A core of genes that are highly transcribed almost exclusively in late-fed ticks (FED5d) was identified (Fig. 1c). Upon closer examination, some of these genes are related to the “secreted” functional class, comprising classes commonly identified in tick salivary glands, such as metalloproteases (M12B and M13 families), lipocalins, protease inhibitors (including Kunitz-type and trypsin inhibitor-like), and mucins^9^. High expression of genes belonging to these functional classes was observed both in UF and FED5d; however, a switch of genes between the groups was observed (Supplementary Fig. 2), suggesting a strategy to alter the molecular repertoire needed for the subsequent blood meal.

Previous studies with *Ornithodoros moubata* (Murray) and *Ornithodoros erraticus* (Lucas) demonstrated higher transcriptional activity of salivary genes at 7 and 14 days after feeding compared to the unfed groups^14,15^. Interestingly, findings observed here for the late-fed group align with these results. In rapid feeders, unlike the genes related to blood meal processing, salivary proteins must be already translated and stored in the salivary glands, allowing ticks to feed as soon as they encounter a vertebrate host. This suggests that *Ornithodoros* ticks resume the transcription of salivary genes within a couple of days after a blood feeding, therefore, returning to a basal condition in preparation for a subsequent blood meal. Because the present study was based solely on a transcriptomic approach, limitations regarding turnover between mRNA synthesis and protein translation should be considered. Nevertheless, it has been previously reported by proteomics that these classes of proteins (*i*.*e*., lipocalins, protease inhibitors, metalloproteases, among others) are already present in the saliva of unfed *O. moubata*^39,40^. However, we should interpret these findings with caution, since our study relies exclusively on whole-body transcriptome data, and the validation of whether these transcripts are, in fact, of salivary gland origin must be confirmed.

An intriguing aspect of soft tick feeding is the uncertainty surrounding the ability to alter the expression of salivary components during the feeding process. This phenomenon, known as “sialome switching”, has been widely documented for hard ticks^41–43^. This mechanism is believed to facilitate evasion of the host's immune system and enables hard ticks to feed uninterruptedly over long-term periods. However, it might be reasonable to assume the absence of “sialome switching” in *Ornithodoros* ticks. They feed rapidly, do not produce cement (a substance secreted that enables ticks to fixate and fasten the mouthparts to the host skin), and might not overcome a short-term host’s defense against their saliva. Hence, it is most likely that soft ticks approach a “one-shot sialome” strategy, featuring efficient and potent anti-clotting, anti-platelet, and vasodilatory components, similar to what is observed in other hematophagous arthropods, besides hard ticks^1^. Indeed, an extensive subcutaneous hemorrhage caused by *O*. *hermsi* saliva may underpin this hypothesis (as discussed below). Potent saliva, destroying cellular components (including immune cells) at the bite site, along with potent anti-coagulant and painkiller components, might guarantee the blood pool formation and blood meal acquisition.

On the other hand, considering that *Ornithodoros* ticks initiate gene transcription of classical classes of salivary genes at least 5 days after feeding (as per our present study) and maintain this activity for 7–14 days^14,15^; the question arises as to whether different genes or isoforms might be transcribed for subsequent feedings, akin to an “off-host sialome switching” phenomenon. In fact, in their natural habitat, *O*. *hermsi* ticks infest the nests and burrows of small rodents (e.g., chipmunks) and may repeatedly feed on the same host across different stages and/or subsequent gonotrophic cycles (for adults)^7,8^. Therefore, they likely developed strategies to evade the immune response of recurrently bitten hosts.

### Insights into the bite of *O. hermsi*

**Figure 4:**
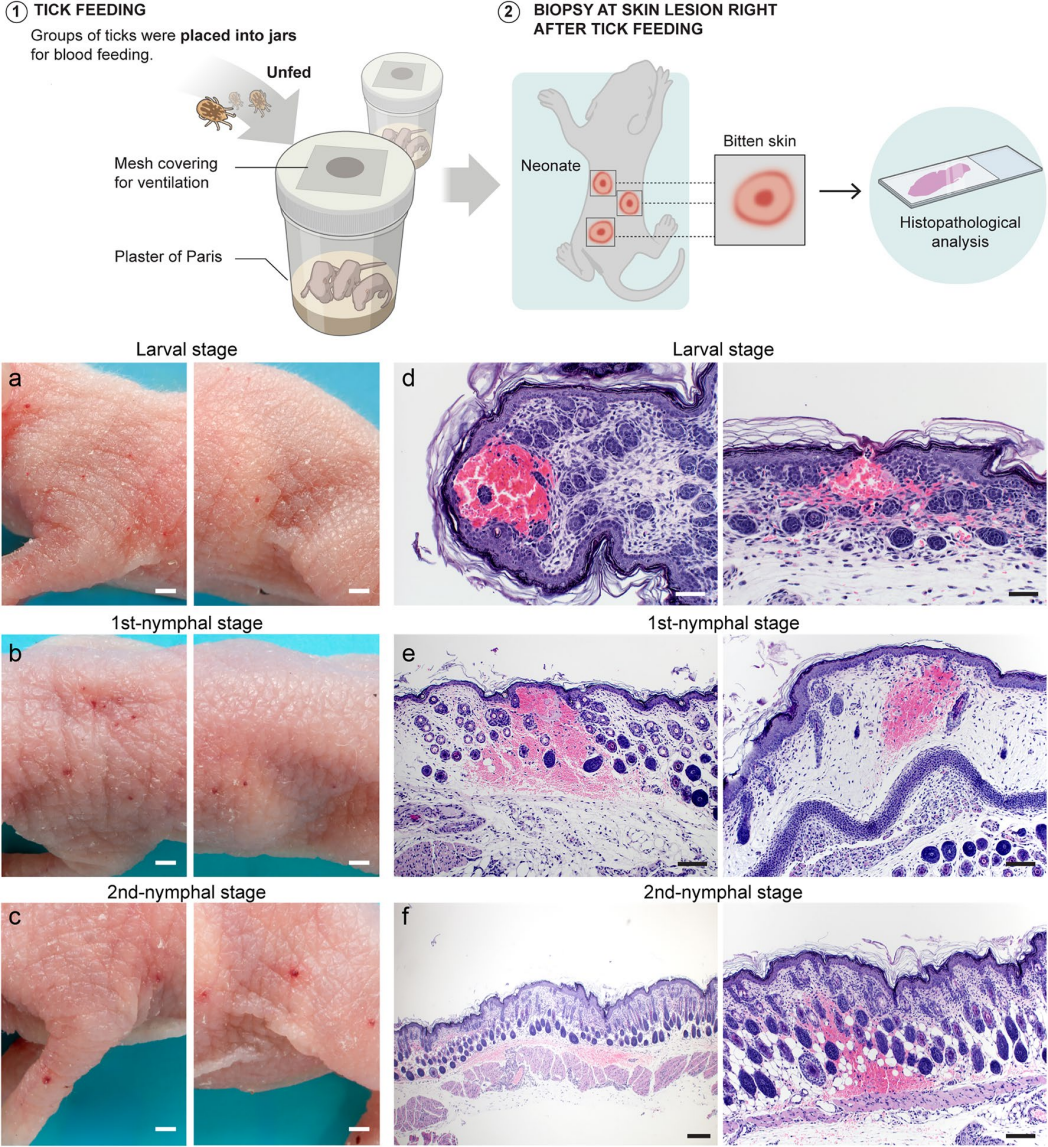
Effect of bites from *Ornithodoros hermsi* immature ticks on the host's skin. Ticks were fed on neonate mice until blood repletion. The formation of erythema at the bite site by larvae (**a**), 1st-nymphs (**b**), and 2nd-nymphs (**c**) was observed immediately after tick feeding (bars: 1 mm). Tick bite sites were excised and used for histopathological analysis and stained with hematoxylin–eosin. Subcutaneous hemorrhage was the primary finding in the skin bitten by *O*. *hermsi* larvae (**d**; 200 × magnification, bar: 50 µm), 1st-nymphs (**e**; 100 × magnification, bar: 100 µm), and 2nd-nymphs (**f**; left, 40 × magnification, bar: 200 µm; right, 100 × magnification, bar: 100 µm).

We observed that upon contact with neonates, *O. hermsi* ticks swiftly attached themselves and commenced feeding. The aftermath of their feeding process was marked by the appearance of a notable hemorrhagic focus at the tick bite site. A detailed histopathological analysis further elucidated the consequences of *O. hermsi* tick bites, revealing evidence of extensive subcutaneous hemorrhage (Fig. 4). These findings of skin hemorrhage are described in the bites of other soft tick species^44^, contrasting with the skin histology of hosts bitten by hard ticks^45–47^. These histologic responses demonstrate different strategies employed by hard and soft ticks to acquire a blood meal.

**Figure 5:**
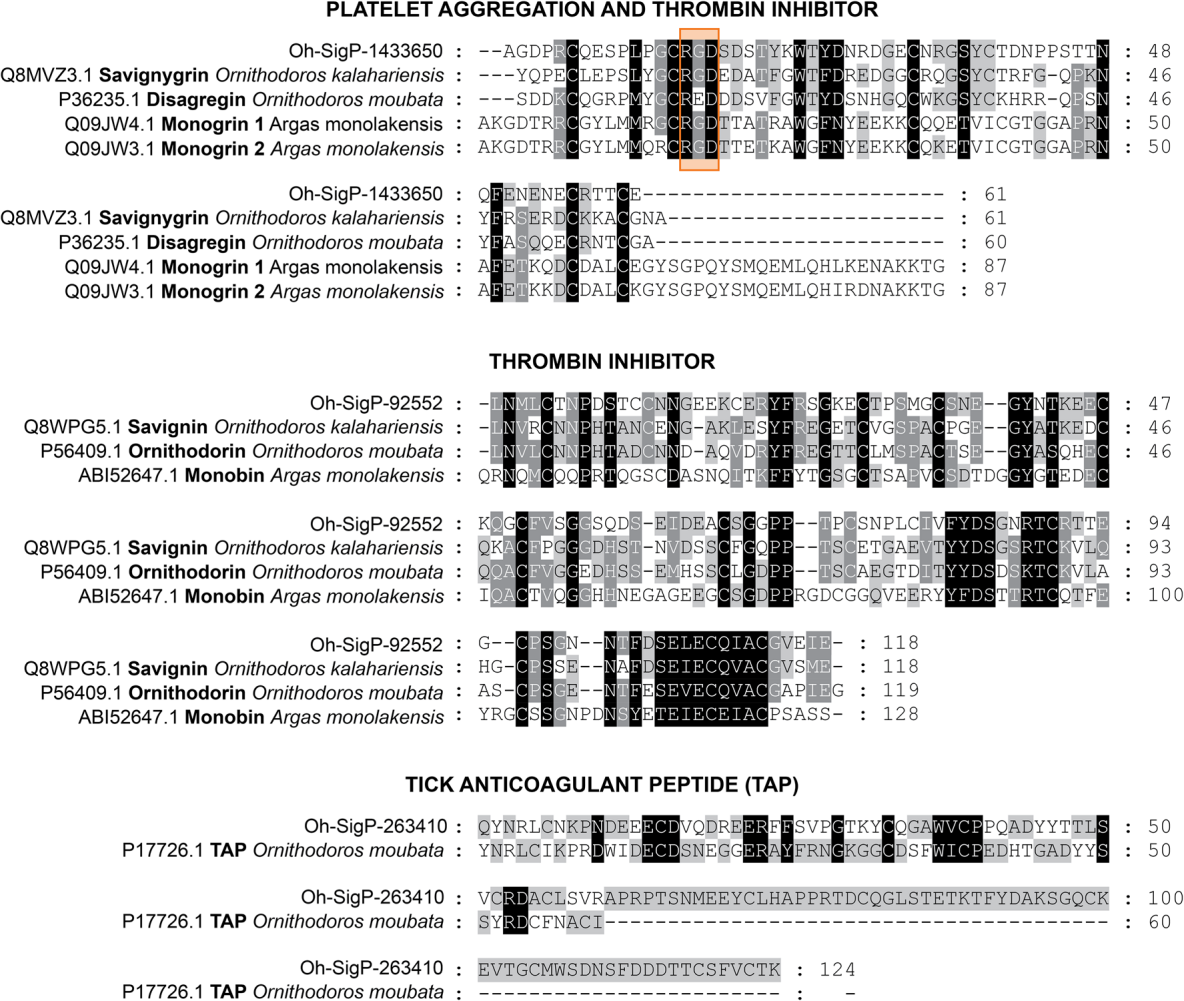
Putative anti-clotting-related proteins found in *Ornithodoros hermsi* transcriptome. ClustalW sequence alignment showing amino acid similarity among *O*. *hermsi* transcripts and characterized anti-clotting proteins from different soft tick species. Orange box highlights the RGD domain. GenBank accession codes are provided before sequences’ names.

Hemorrhagic skin lesions induced by *O. hermsi* bites may result from the presence of proteolytic enzymes and potent anti-hemostatic agents in the saliva of this tick species. Indeed, the *O. hermsi* transcriptome is a rich source of proteases from different families, including metalloproteases (families M12B and M13) and serine-proteases (family S01A) (see Supplementary File 1 and Supplementary File 2). In soft ticks, anti-clotting mechanisms have been described for the genus *Ornithodoros* and *Argas*, including factor Xa, thrombin, and platelet aggregation inhibitors^48–51^. Homologs of the tick anticoagulant peptide (TAP)^51^, savignygrin^52^, disagregin^53^, monogrin^49^, savignin^48^, and ornithodorin^54^ were identified in this study (Fig. 5). These homologs in *O. hermsi* shows its expression peaking at the UF and/or FED5d after feeding in both developmental stages analyzed here (Supplementary Fig. 2). Although homologs to these inhibitors previously isolated from salivary glands of other soft tick species have been identified in the whole-body transcriptome of *O. hermsi*, additional studies are needed to demonstrate that these transcripts are indeed expressed in the salivary glands.

Systemic disturbances in the coagulation system have been reported in animals bitten by *Ornithodoros brasiliensis* (Aragão)^44^. In addition, the bites of soft ticks are acknowledged to produce the most variable symptoms, including paralysis, toxicosis, fever, pain, pruritus, among other manifestations^44,55–60^. A few studies evaluated the effect of *O*. *hermsi* bites on the host, including human beings^8,61^. These reports suggest that its bites appear to be quite benign, causing little discomfort to the host. Although further investigations on *O*. *hermsi* bite effect are needed, given the observed injury at the bite site, it is prudent to suggest that *O*. *hermsi* saliva also contains yet-to-be-discovered molecules influencing the perception of pain in the host. Altogether, these findings emphasize the importance of understanding the feeding biology, contributing to our broader comprehension of the interactions between soft ticks and their vertebrate hosts.

## Conclusion

In summary, this study contributes a substantial repertoire of transcripts for a medically relevant soft tick species with relatively limited prior knowledge. Importantly, our results demonstrate higher transcriptional activity in unfed *O*. *hermsi* ticks compared to the fed groups at various time points after detachment. These suggest that unfed ticks intensify the transcription of genes, which are crucial for blood feeding and digestion prior to the blood meal, potentially explaining the rapid feeding strategy generally adopted by the Argasidae family. Our histopathological analysis revealed an extensive subcutaneous hemorrhage caused by *O*. *hermsi* bites, likely trigged by tick salivary components. Altogether, these results offer a glimpse into the strategies that *Ornithodoros* ticks employ for hematophagy.

### Supplementary Information

Supplementary Figure 1.

Supplementary Figure 2.

Supplementary Information 3.

Supplementary Information 4.

Supplementary Information 5.

Supplementary Legends.

## Data Availability

The transcriptome data have been deposited in the National Center for Biotechnology Information (NCBI) under BioProject PRJNA911751 with Biosamples SAMN32192832, SAMN32192833, SAMN32192834, SAMN32192835, SAMN32192836, SAMN32192837, SAMN32192838, SAMN32192839, SAMN32192840, SAMN32192841, SAMN32192842, SAMN32192843, SAMN32192844, SAMN32192845, SAMN32192846, SAMN32370292, SAMN32370293, SAMN32370294, SAMN32370295, SAMN32370296, SAMN32370297, SAMN32370298, SAMN32370299, SAMN32370300, SAMN32370301, SAMN32370302, SAMN32370303, SAMN32370304, SAMN32370305, SAMN32370306, SAMN32370311, SAMN32370312, SAMN32370313, SAMN32370314, SAMN32370315, SAMN32370316, SAMN32370317, SAMN32370318, SAMN32370319, SAMN32370320, SAMN32370321, SAMN32370322, SAMN32370323, SAMN32370324, SAMN32370325. The raw reads have been submitted to the Sequence Reads Archives (SRA) under accessions SRR22868584, SRR22868586, SRR22868580, SRR22868582, SRR22868577, SRR22868583, SRR22868576, SRR22868574, SRR22868575, SRR22868587, SRR22868573, SRR22868581, SRR22868579, SRR22868578, SRR22868585, SRR22868572, SRR22868568, SRR22868569, SRR22868564, SRR22868562, SRR22868561, SRR22868571, SRR22868567, SRR22868560, SRR22868563, SRR22868566, SRR22868559, SRR22868558, SRR22868565, SRR22868570, SRR22738886, SRR22738882, SRR22738888, SRR22738890, SRR22738880, SRR22738893, SRR22738894, SRR22738881, SRR22738892, SRR22738889, SRR22738887, SRR22738891, SRR22738883, SRR22738885, SRR22738884. This Transcriptome Shotgun Assembly project has been deposited at DDBJ/EMBL/GenBank under the accession GKRQ00000000. The version described in this paper is the first version, GKRQ01000000.
