# Supplementary figures and images for: Exploring the transcriptome of immature stages of Ornithodoros hermsi, the soft-tick vector of tick-borne relapsing fever
Source: Sci Rep. 2024 May 30;14:12466. doi: 10.1038/s41598-024-62732-6 (PMC11140000; doi:10.1038/s41598-024-62732-6)

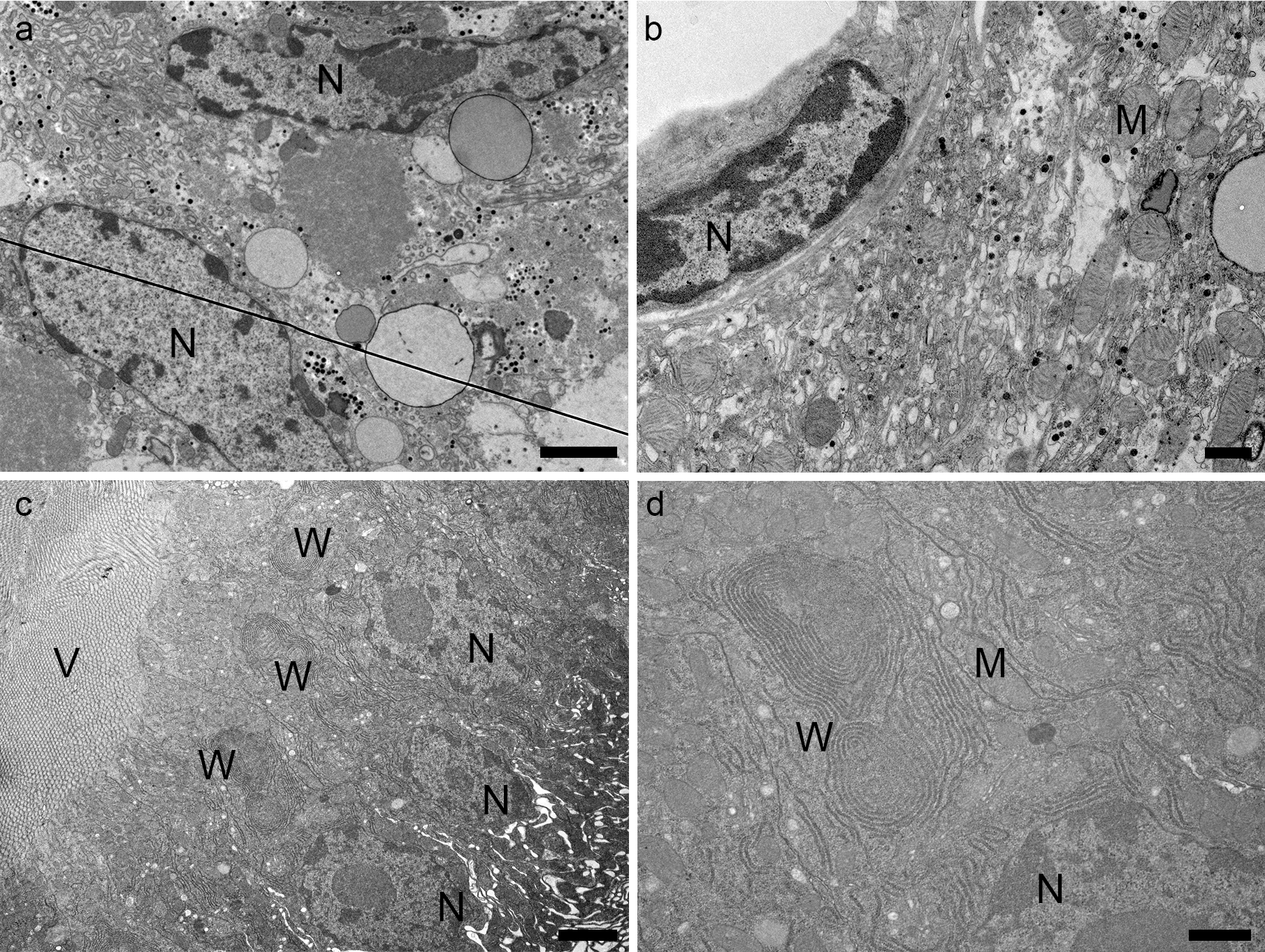

Supplement: Supplementary file 1 — Supplementary Figure 1. [file 41598_2024_62732_MOESM1_ESM.tif]
