# Supplementary material for: Exploring the transcriptome of immature stages of Ornithodoros hermsi, the soft-tick vector of tick-borne relapsing fever
Source: Sci Rep. 2024 May 30;14:12466. doi: 10.1038/s41598-024-62732-6 (PMC11140000; doi:10.1038/s41598-024-62732-6)
Supplement: Supplementary file 2 — Supplementary Figure 2. [file 41598_2024_62732_MOESM2_ESM.pdf]

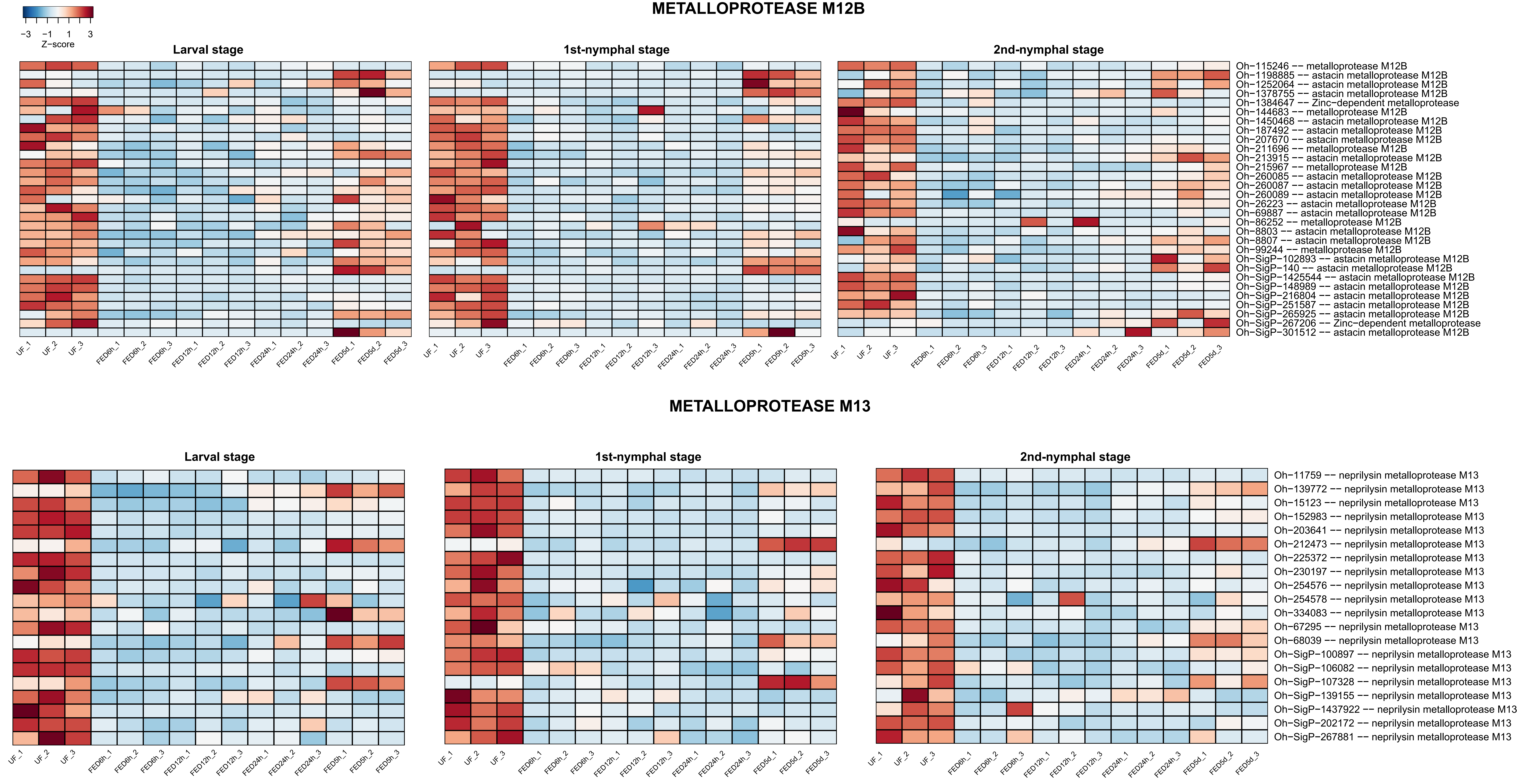
METALLOPROTEASE M12B
−3 −1 1 3
Z−score
Larval stage
1st-nymphal stage
2nd-nymphal stage
Oh−115246 −− metalloprotease M12B
Oh−1198885 −− astacin metalloprotease M12B
Oh−1252064 −− astacin metalloprotease M12B
Oh−1378755 −− astacin metalloprotease M12B
Oh−1384647 −− Zinc−dependent metalloprotease
Oh−144683 −− metalloprotease M12B
Oh−1450468 −− astacin metalloprotease M12B
Oh−187492 −− astacin metalloprotease M12B
Oh−207670 −− astacin metalloprotease M12B
Oh−211696 −− metalloprotease M12B
Oh−213915 −− astacin metalloprotease M12B
Oh−215967 −− metalloprotease M12B
Oh−260085 −− astacin metalloprotease M12B
Oh−260087 −− astacin metalloprotease M12B
Oh−260089 −− astacin metalloprotease M12B
Oh−26223 −− astacin metalloprotease M12B
Oh−69887 −− astacin metalloprotease M12B
Oh−86252 −− metalloprotease M12B
Oh−8803 −− astacin metalloprotease M12B
Oh−8807 −− astacin metalloprotease M12B
Oh−99244 −− metalloprotease M12B
Oh−SigP−102893 −− astacin metalloprotease M12B
Oh−SigP−140 −− astacin metalloprotease M12B
Oh−SigP−1425544 −− astacin metalloprotease M12B
Oh−SigP−148989 −− astacin metalloprotease M12B
Oh−SigP−216804 −− astacin metalloprotease M12B
Oh−SigP−251587 −− astacin metalloprotease M12B
Oh−SigP−265925 −− astacin metalloprotease M12B
Oh−SigP−267206 −− Zinc−dependent metalloprotease
Oh−SigP−301512 −− astacin metalloprotease M12B
UF_1 UF_2 UF_3 FED6h_1 FED6h_2 FED6h_3 FED12h_1 FED12h_2 FED12h_3 FED24h_1 FED24h_2 FED24h_3 FED5d_1 FED5d_2 FED5d_3
UF_1 UF_2 UF_3 FED6h_1 FED6h_2 FED6h_3 FED12h_1 FED12h_2 FED12h_3 FED24h_1 FED24h_2 FED24h_3 FED5h_1 FED5h_2 FED5h_3
UF_1 UF_2 UF_3 FED6h_1 FED6h_2 FED6h_3 FED12h_1 FED12h_2 FED12h_3 FED24h_1 FED24h_2 FED24h_3 FED5d_1 FED5d_2 FED5d_3
METALLOPROTEASE M13
Larval stage
1st-nymphal stage
2nd-nymphal stage
Oh−11759 −− neprilysin metalloprotease M13
Oh−139772 −− neprilysin metalloprotease M13
Oh−15123 −− neprilysin metalloprotease M13
Oh−152983 −− neprilysin metalloprotease M13
Oh−203641 −− neprilysin metalloprotease M13
Oh−212473 −− neprilysin metalloprotease M13
Oh−225372 −− neprilysin metalloprotease M13
Oh−230197 −− neprilysin metalloprotease M13
Oh−254576 −− neprilysin metalloprotease M13
Oh−254578 −− neprilysin metalloprotease M13
Oh−334083 −− neprilysin metalloprotease M13
Oh−67295 −− neprilysin metalloprotease M13
Oh−68039 −− neprilysin metalloprotease M13
Oh−SigP−100897 −− neprilysin metalloprotease M13
Oh−SigP−106082 −− neprilysin metalloprotease M13
Oh−SigP−107328 −− neprilysin metalloprotease M13
Oh−SigP−139155 −− neprilysin metalloprotease M13
Oh−SigP−1437922 −− neprilysin metalloprotease M13
Oh−SigP−202172 −− neprilysin metalloprotease M13
Oh−SigP−267881 −− neprilysin metalloprotease M13
UF_1 UF_2 UF_3 FED6h_1 FED6h_2 FED6h_3 FED12h_1 FED12h_2 FED12h_3 FED24h_1 FED24h_2 FED24h_3 FED5h_1 FED5h_2 FED5h_3
UF_1 UF_2 UF_3 FED6h_1 FED6h_2 FED6h_3 FED12h_1 FED12h_2 FED12h_3 FED24h_1 FED24h_2 FED24h_3 FED5d_1 FED5d_2 FED5d_3
UF_1 UF_2 UF_3 FED6h_1 FED6h_2 FED6h_3 FED12h_1 FED12h_2 FED12h_3 FED24h_1 FED24h_2 FED24h_3 FED5d_1 FED5d_2 FED5d_3

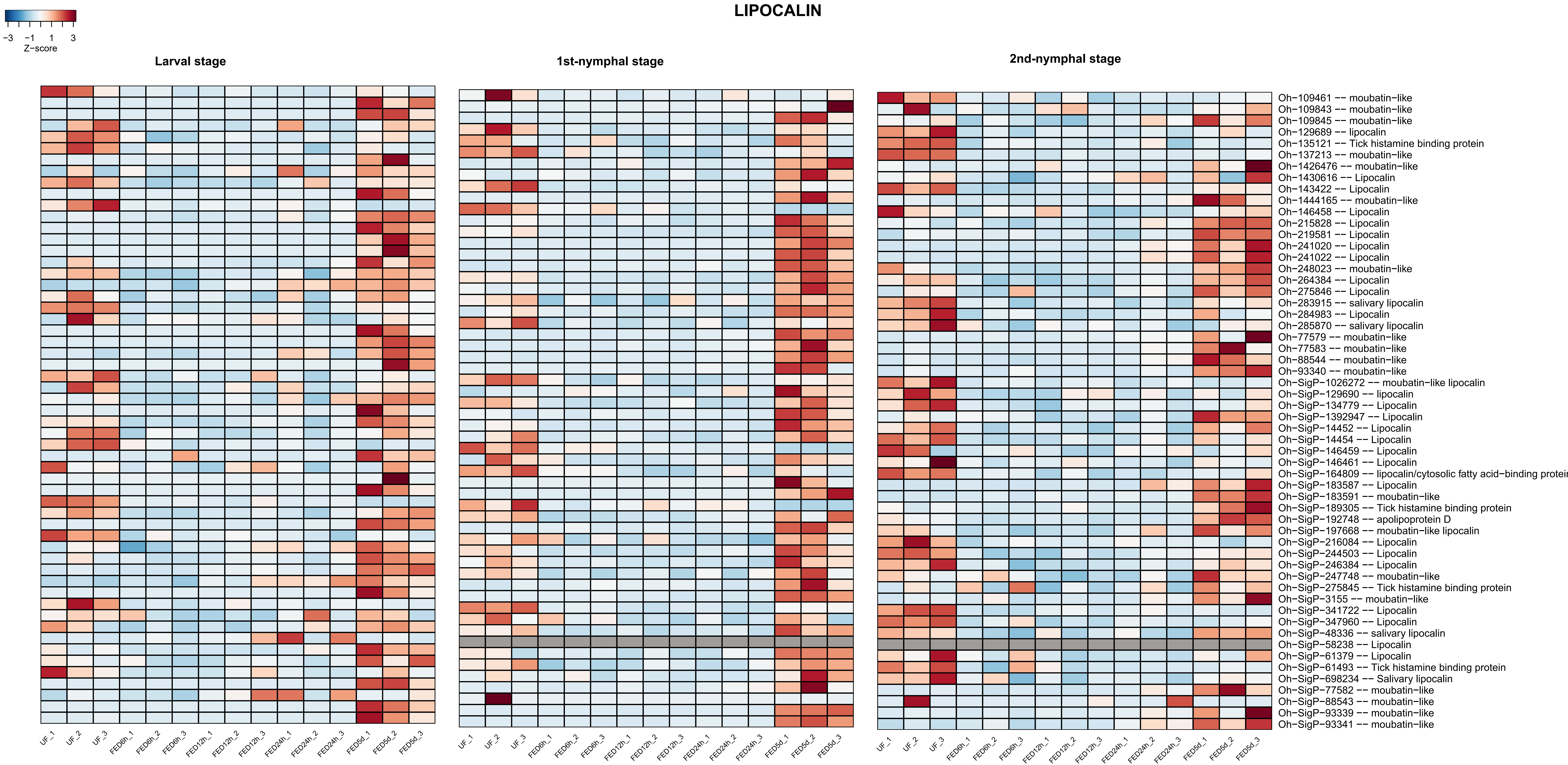
LIPOCALIN
−3 −1 1 3
Z−score
Larval stage
1st-nymphal stage
2nd-nymphal stage
UF_1
UF_2
UF_3
FED6h_1
FED6h_2
FED6h_3
FED12h_1
FED12h_2
FED12h_3
FED24h_1
FED24h_2
FED24h_3
FED5d_1
FED5d_2
FED5d_3
Oh−109461 −− moubatin−like
Oh−109843 −− moubatin−like
Oh−109845 −− moubatin−like
Oh−129689 −− lipocalin
Oh−135121 −− Tick histamine binding protein
Oh−137213 −− moubatin−like
Oh−1426476 −− moubatin−like
Oh−1430616 −− Lipocalin
Oh−143422 −− Lipocalin
Oh−1444165 −− moubatin−like
Oh−146458 −− Lipocalin
Oh−215828 −− Lipocalin
Oh−219581 −− Lipocalin
Oh−241020 −− Lipocalin
Oh−241022 −− Lipocalin
Oh−248023 −− moubatin−like
Oh−264384 −− Lipocalin
Oh−275846 −− Lipocalin
Oh−283915 −− salivary lipocalin
Oh−284983 −− Lipocalin
Oh−285870 −− salivary lipocalin
Oh−77579 −− moubatin−like
Oh−77583 −− moubatin−like
Oh−88544 −− moubatin−like
Oh−93340 −− moubatin−like
Oh−SigP−1026272 −− moubatin−like lipocalin
Oh−SigP−129690 −− lipocalin
Oh−SigP−134779 −− Lipocalin
Oh−SigP−1392947 −− Lipocalin
Oh−SigP−14452 −− Lipocalin
Oh−SigP−14454 −− Lipocalin
Oh−SigP−146459 −− Lipocalin
Oh−SigP−146461 −− Lipocalin
Oh−SigP−164809 −− lipocalin/cytosolic fatty acid−binding protei
Oh−SigP−183587 −− Lipocalin
Oh−SigP−183591 −− moubatin−like
Oh−SigP−189305 −− Tick histamine binding protein
Oh−SigP−192748 −− apolipoprotein D
Oh−SigP−197668 −− moubatin−like lipocalin
Oh−SigP−216084 −− Lipocalin
Oh−SigP−244503 −− Lipocalin
Oh−SigP−246384 −− Lipocalin
Oh−SigP−247748 −− moubatin−like
Oh−SigP−275845 −− Tick histamine binding protein
Oh−SigP−3155 −− moubatin−like
Oh−SigP−341722 −− Lipocalin
Oh−SigP−347960 −− Lipocalin
Oh−SigP−48336 −− salivary lipocalin
Oh−SigP−58238 −− Lipocalin
Oh−SigP−61379 −− Lipocalin
Oh−SigP−61493 −− Tick histamine binding protein
Oh−SigP−698234 −− Salivary lipocalin
Oh−SigP−77582 −− moubatin−like
Oh−SigP−88543 −− moubatin−like
Oh−SigP−93339 −− moubatin−like
Oh−SigP−93341 −− moubatin−like

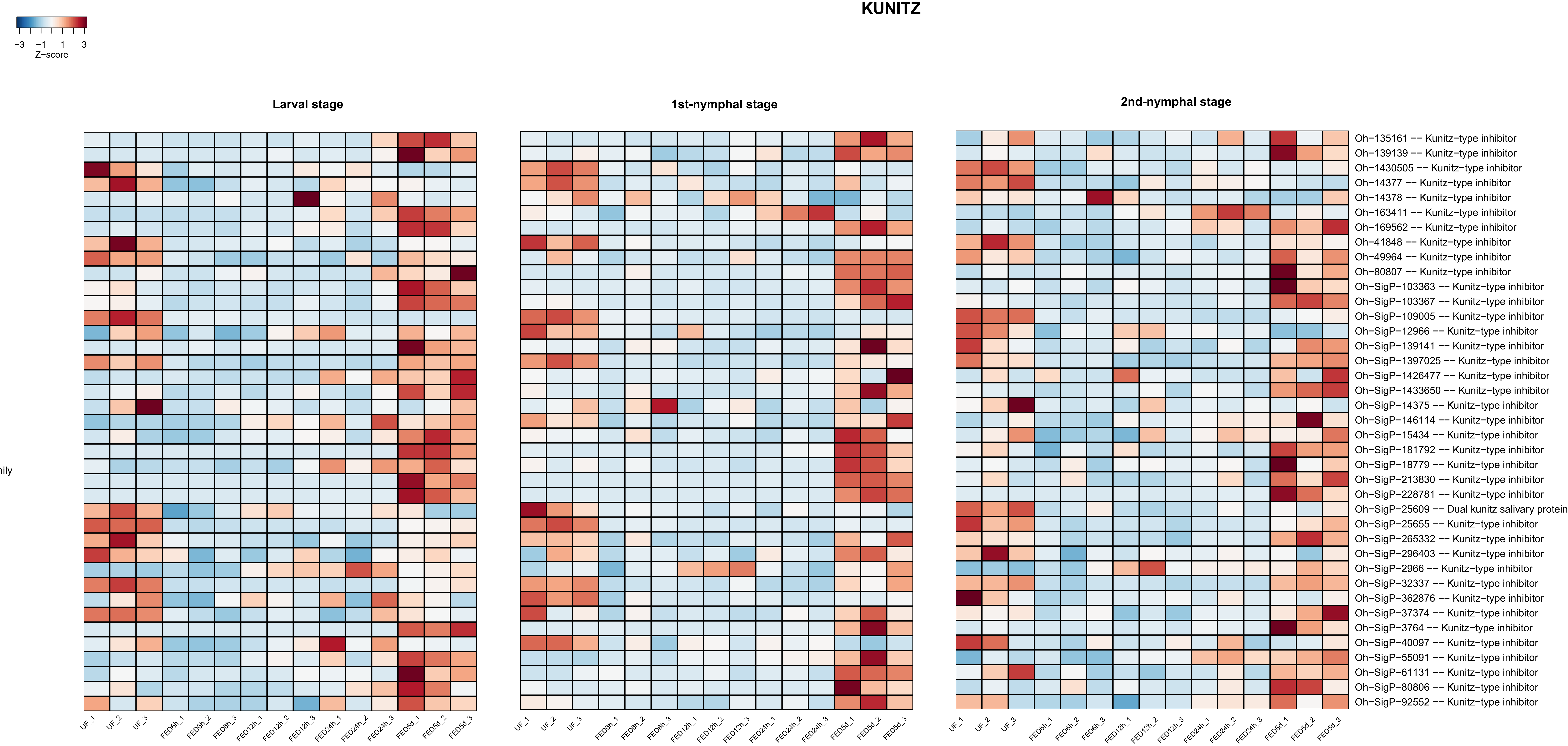
KUNITZ
−3 −1 1 3
Z−score
Larval stage
1st-nymphal stage
2nd-nymphal stage
Oh−135161 −− Kunitz−type inhibitor
Oh−139139 −− Kunitz−type inhibitor
Oh−1430505 −− Kunitz−type inhibitor
Oh−14377 −− Kunitz−type inhibitor
Oh−14378 −− Kunitz−type inhibitor
Oh−163411 −− Kunitz−type inhibitor
Oh−169562 −− Kunitz−type inhibitor
Oh−41848 −− Kunitz−type inhibitor
Oh−49964 −− Kunitz−type inhibitor
Oh−80807 −− Kunitz−type inhibitor
Oh−SigP−103363 −− Kunitz−type inhibitor
Oh−SigP−103367 −− Kunitz−type inhibitor
Oh−SigP−109005 −− Kunitz−type inhibitor
Oh−SigP−12966 −− Kunitz−type inhibitor
Oh−SigP−139141 −− Kunitz−type inhibitor
Oh−SigP−1397025 −− Kunitz−type inhibitor
Oh−SigP−1426477 −− Kunitz−type inhibitor
Oh−SigP−1433650 −− Kunitz−type inhibitor
Oh−SigP−14375 −− Kunitz−type inhibitor
Oh−SigP−146114 −− Kunitz−type inhibitor
Oh−SigP−15434 −− Kunitz−type inhibitor
Oh−SigP−181792 −− Kunitz−type inhibitor
Oh−SigP−18779 −− Kunitz−type inhibitor
Oh−SigP−213830 −− Kunitz−type inhibitor
Oh−SigP−228781 −− Kunitz−type inhibitor
Oh−SigP−25609 −− Dual kunitz salivary protein
Oh−SigP−25655 −− Kunitz−type inhibitor
Oh−SigP−265332 −− Kunitz−type inhibitor
Oh−SigP−296403 −− Kunitz−type inhibitor
Oh−SigP−2966 −− Kunitz−type inhibitor
Oh−SigP−32337 −− Kunitz−type inhibitor
Oh−SigP−362876 −− Kunitz−type inhibitor
Oh−SigP−37374 −− Kunitz−type inhibitor
Oh−SigP−3764 −− Kunitz−type inhibitor
Oh−SigP−40097 −− Kunitz−type inhibitor
Oh−SigP−55091 −− Kunitz−type inhibitor
Oh−SigP−61131 −− Kunitz−type inhibitor
Oh−SigP−80806 −− Kunitz−type inhibitor
Oh−SigP−92552 −− Kunitz−type inhibitor
UF_1
UF_2
UF_3
FED6h_1
FED6h_2
FED6h_3
FED12h_1
FED12h_2
FED12h_3
FED24h_1
FED24h_2
FED24h_3
FED5d_1
FED5d_2
FED5d_3

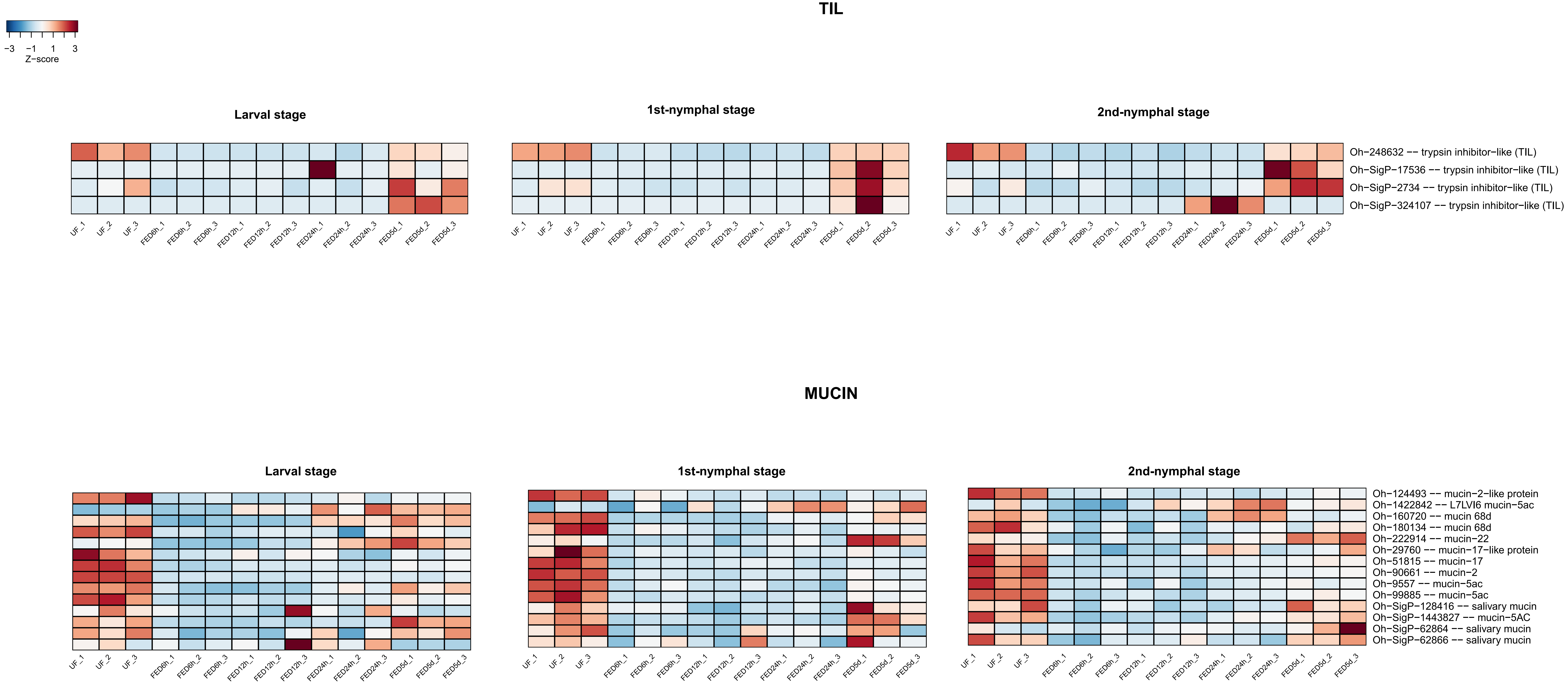

TIL
−3 −1 1 3
Z−score
Larval stage
1st-nymphal stage
2nd-nymphal stage
Oh−248632 −− trypsin inhibitor−like (TIL)
Oh−SigP−17536 −− trypsin inhibitor−like (TIL)
Oh−SigP−2734 −− trypsin inhibitor−like (TIL)
Oh−SigP−324107 −− trypsin inhibitor−like (TIL)
UF_1 UF_2 UF_3 FED6h_1 FED6h_2 FED6h_3 FED12h_1 FED12h_2 FED12h_3 FED24h_1 FED24h_2 FED24h_3 FED5d_1 FED5d_2 FED5d_3
MUCIN
Larval stage
1st-nymphal stage
2nd-nymphal stage
Oh−124493 −− mucin−2−like protein
Oh−1422842 −− L7LVI6 mucin−5ac
Oh−160720 −− mucin 68d
Oh−180134 −− mucin 68d
Oh−222914 −− mucin−22
Oh−29760 −− mucin−17−like protein
Oh−51815 −− mucin−17
Oh−90661 −− mucin−2
Oh−9557 −− mucin−5ac
Oh−99885 −− mucin−5ac
Oh−SigP−128416 −− salivary mucin
Oh−SigP−1443827 −− mucin−5AC
Oh−SigP−62864 −− salivary mucin
Oh−SigP−62866 −− salivary mucin
UF_1 UF_2 UF_3 FED6h_1 FED6h_2 FED6h_3 FED12h_1 FED12h_2 FED12h_3 FED24h_1 FED24h_2 FED24h_3 FED5d_1 FED5d_2 FED5d_3
