# Supplementary material for: Exploring the transcriptome of immature stages of Ornithodoros hermsi, the soft-tick vector of tick-borne relapsing fever
Source: Sci Rep. 2024 May 30;14:12466. doi: 10.1038/s41598-024-62732-6 (PMC11140000; doi:10.1038/s41598-024-62732-6)
Supplement: Supplementary file 3 — Supplementary Information 3. [file 41598_2024_62732_MOESM3_ESM.docx]

**Supplementary File 1:** A Windows-compatible hyperlinked Excel file that includes functional annotation for all 103,646 coding sequences identified in this study. This file can be downloaded as a single .zip file from the following link: <https://proj-bip-prod-publicread.s3.amazonaws.com/transcriptome/O_hermsi/Table-S1.zip>
