# Supplementary material for: Exploring the transcriptome of immature stages of Ornithodoros hermsi, the soft-tick vector of tick-borne relapsing fever
Source: Sci Rep. 2024 May 30;14:12466. doi: 10.1038/s41598-024-62732-6 (PMC11140000; doi:10.1038/s41598-024-62732-6)
Supplement: Supplementary file 4 — Supplementary Information 4. [file 41598_2024_62732_MOESM4_ESM.docx]

**Supplementary File 2:** A Windows-compatible hyperlinked Excel file that includes functional annotation for all 18,465 coding sequences identified in this study meeting the expression threshold of a TMP≥5. This file can be downloaded as a single .zip file from the following link:

<https://proj-bip-prod-publicread.s3.amazonaws.com/transcriptome/O_hermsi/Table+S2.zip>
