# Supplementary material for: Exploring the transcriptome of immature stages of Ornithodoros hermsi, the soft-tick vector of tick-borne relapsing fever
Source: Sci Rep. 2024 May 30;14:12466. doi: 10.1038/s41598-024-62732-6 (PMC11140000; doi:10.1038/s41598-024-62732-6)
Supplement: Supplementary file 6 — Supplementary Legends. [file 41598_2024_62732_MOESM6_ESM.docx]

**Supplementary Information**

**Supplementary File 1:** A Windows-compatible hyperlinked Excel file that includes functional annotation for all 103,646 coding sequences identified in this study. This file can be downloaded as a single .zip file from the following link: <https://proj-bip-prod-publicread.s3.amazonaws.com/transcriptome/O_hermsi/Table-S1.zip>

**Supplementary File 2:** A Windows-compatible hyperlinked Excel file that includes functional annotation for all 18,465 coding sequences identified in this study meeting the expression threshold of a TMP≥5. This file can be downloaded as a single .zip file from the following link:

<https://proj-bip-prod-publicread.s3.amazonaws.com/transcriptome/O_hermsi/Table+S2.zip>

**Supplementary File 3:** Functional classification of the transcripts of immature *Ornithodoros hermsi* ticks.

**Supplementary Figure 1: Electron microscopy of unfed *Ornithodoros hermsi* midgut epithelial cells.**

*Ornithodoros hermsi* midguts were collected and analyzed using electron microscopy. Whorls-like endoplasmic reticulum was not observed in unfed *O*. *hermsi* 1^st^-nymphal stage midgut epithelial cells (**a**, bar: 2 µm; and **b**, bar: 1 µm). As a control, we analyzed midguts of unfed *Aedes* *aegypti* mosquito midgut epithelial cells. Whorls were clearly visible in mosquito midguts (**c**, bar: 2 µm; and **d**, bar: 1 µm).

Note: N = nucleus; M = mitochondria; W: whorls-like endoplasmic reticulum.

**Supplementary Figure 2: Overview of the transcriptional profile of putative salivary transcripts of *Ornithodoros hermsi* immature stages across different feeding timepoints.**

Heatmap plots compare the relative abundance (TPM ≥ 5 and transformed into Z-score) of transcripts between feeding groups.
